# Migratory Fish Bone in the Hyoglossus: Catch Me if You Can

**DOI:** 10.7759/cureus.58215

**Published:** 2024-04-13

**Authors:** Muhammad Adzha Musa, Irfan Affandi Hamid, Hardip Gendeh

**Affiliations:** 1 Department of Otorhinolaryngology-Head and Neck Surgery, Hospital Canselor Tuanku Muhriz, Kuala Lumpur, MYS; 2 Department of Otorhinolaryngology-Head and Neck Surgery, Hospital Sultanah Aminah, Johor Bahru, MYS

**Keywords:** pharyngeal foreign body, foreign body removal, ingested foreign body, migratory fishbone, fish bone

## Abstract

Foreign body ingestion is one of the most frequently encountered cases in otorhinolaryngology and most of the cases can be managed non-operatively. If left untreated, migration of foreign bodies can occur and presents a significant challenge in patient management. We hereby describe the case of an elderly gentleman who had a preceding history of fish bone ingestion and complained of dysphagia for two days. Clinical examination revealed swelling of the right vallecula with minimal pus discharge. Computed tomography (CT) of the neck confirmed the diagnosis of a migratory foreign body in the neck. He underwent open neck exploration and foreign body removal under intraoperative fluoroscopy guidance. A high index of suspicion of a migratory foreign body is warranted in cases of persistent, unresolved symptoms with the failure of endoscopic evaluation to detect the foreign body. Migratory foreign body of the neck may cause life-threatening complications and requires early surgical intervention.

## Introduction

Foreign body ingestion is one of the common presentations in otorhinolaryngology practice. It is commonly seen in the pediatric population with the peak incidence between the ages of six months to six years [1]. In adults, foreign body ingestion is usually accidental and it is commonly observed in patients with underlying psychiatric disorders, the elderly, alcohol intoxication, and among prisoners [2,3]. Fishbone is the most common type of foreign body in 85% of cases [4] and it may lodge in the upper aerodigestive tract. Most of foreign body ingestion cases can be managed non-operatively with less than 1% requiring active surgical intervention [5]. However, a small percentage of ingested foreign bodies may migrate extraluminal into the neck and may cause severe complications such as deep neck abscess, mediastinitis, esophageal perforation, and fistula. We describe a challenging case of migratory fish bone complicated with deep neck space abscess requiring an open neck exploration after an unsuccessful intraoral approach and discuss what to do when in doubt and factors associated with a migrating fish bone.

## Case presentation

**Figure 1:**
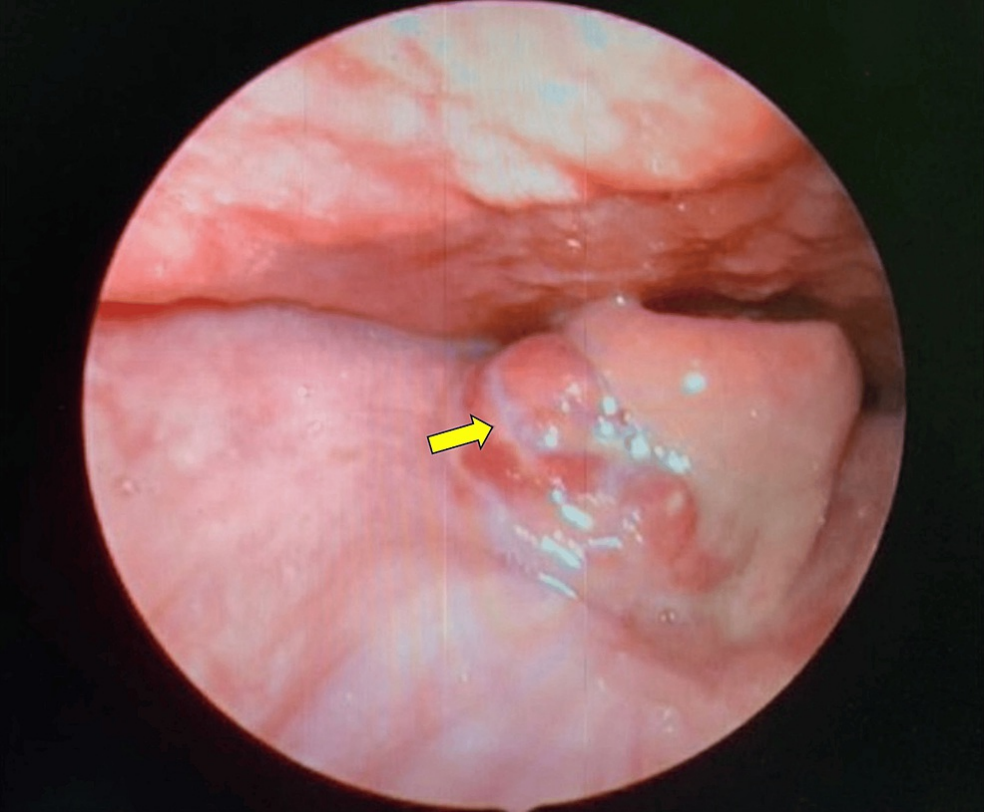
Video laryngoscopy showing a right vallecula swelling (yellow arrow).

**Figure 2:**
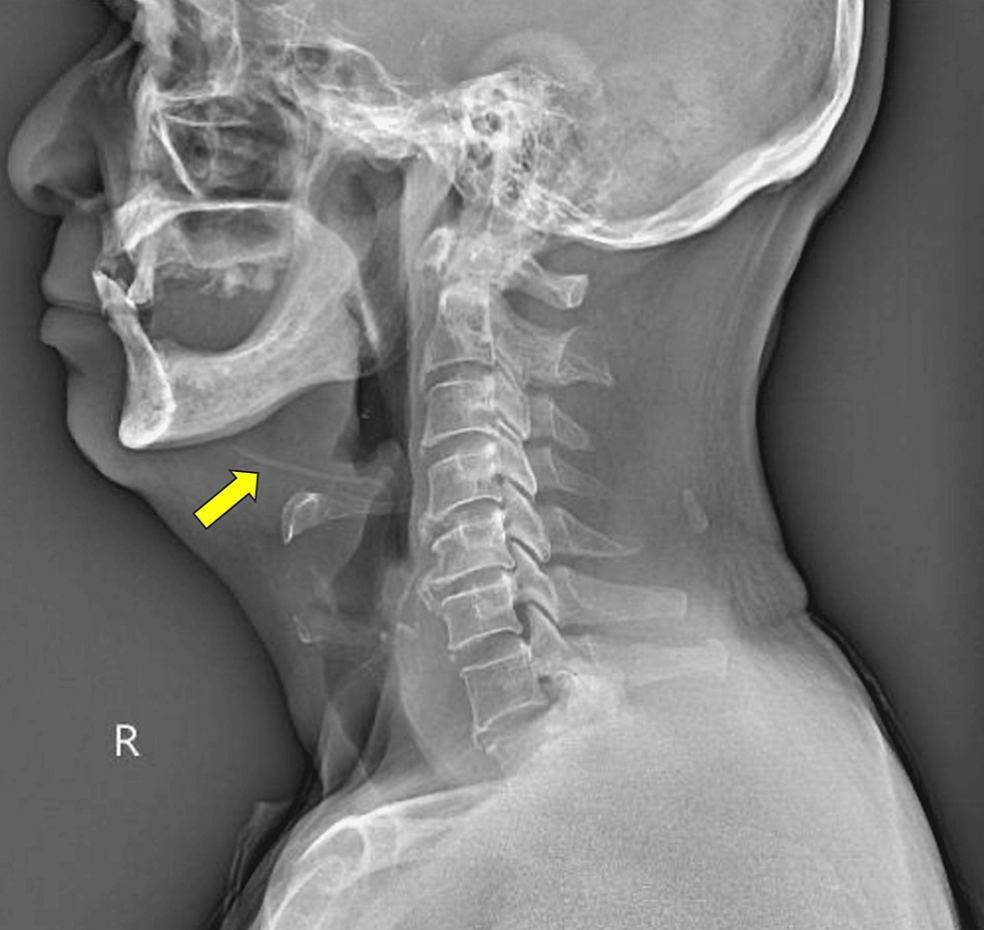
Lateral neck radiograph revealing a linear opacity suggestive of a foreign body within the anterior soft tissue of the neck at the level of the third cervical vertebra (yellow arrow).

A 61-year-old gentleman with hypertension presented with a two-day history of dysphagia. He denied dyspnea, noisy breathing, neck swelling, fever, or a change of voice. He had a preceding history of fish bone ingestion five days prior in which he sought consultation at the Emergency Department. He was discharged as the neck radiograph was normal. Due to unresolved symptoms, he revisited a tertiary hospital for a second opinion. Rigid video laryngoscopy showed swelling over the right vallecula (Figure 1). There was no obvious foreign body or pooling of saliva. A repeated lateral neck radiograph showed a linear opacity suggestive of a foreign body within the anterior soft tissue of the neck at the level of the C3 cervical vertebra (Figure 2).

He underwent direct laryngoscopy, incision, and drainage. Intraoperatively, there was a swelling at the right vallecula with slough. A small incision was made and small amounts of pus were drained. However, there was no foreign bone identified. Subsequently, a contrasted computed tomography (CT) of the neck was done and showed an area of heterogeneous thickening at pre-epiglottic space with ring-enhancing collection measuring 1.3 x 1.2 x 1.1 cm with a hyperdense focus (suggestive of a foreign body) seen anterior to the collection, about 0.8 cm from the right vallecula. The diagnosis was revised to a migratory foreign body with a pre-glottic abscess. He was started on intravenous amoxicillin/clavulanic acid and dexamethasone. He was then referred to the tertiary referral center. 

**Figure 3:**
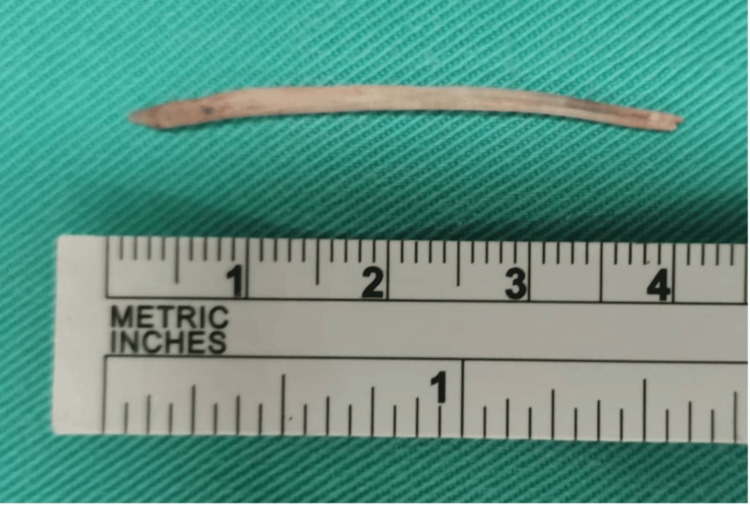
Intra-operative image showing a sharp, linear fish bone measuring about 4 cm.

Upon admission, the patient was immediately scheduled for surgery under general anesthesia. On direct laryngoscopy, the previous incision and drainage site over the right vallecula were explored. There was no foreign body identified and difficulty was encountered in determining the exact location of the foreign body. Intraoperative fluoroscopy was done and the foreign body was identified in the submental region just above the hyoid bone in a slanting position on the lateral view. The neck was explored via a transcervical approach to remove the foreign body. The fishbone measuring about 4 cm in length, embedded inside the hyoglossus muscle, was successfully removed (Figure 3). Postoperatively, the symptoms were markedly improved and a repeated rigid video laryngoscopy on the second day post surgery revealed resolving right vallecula swelling. He completed intravenous antibiotics and dexamethasone for a total of five and three days, respectively, and was discharged well.

## Discussion

The incidence of migratory foreign bodies into the neck space is a rare occurrence and it can occur in any age group. Ingested foreign bodies are commonly lodged in either the palatine tonsil, base of the tongue, pyriform fossa, pharyngeal wall, or cervical esophagus. The theory behind the migration of foreign bodies is due to the contraction of neck muscles and viscera during voluntary and involuntary movements of the head and neck structures. The type of foreign body also influences the migration of foreign bodies. Sharp and linear foreign bodies have been found to have a higher risk of migration [6]. Curved fish bone on the other hand may rotate within its axis and is less likely to migrate. In our case, the foreign was found to be a 4 cm sharp and linear fishbone, near the vallecula.

The ingested foreign bodies that migrate extraluminal pose a serious risk of developing severe complications, depending on the direction and site of migration. Migrated foreign bodies may induce inflammation and bacterial infection in the soft tissue, leading to the formation of deep-space abscesses. The infection may also worsen and spread into the mediastinum, leading to mediastinitis. The foreign body also can penetrate vital adjacent structures such as major vessels in the neck causing carotid rupture, innominate-esophageal fistula, and aorto-esophageal fistula [5-7].

The clinical presentations in migratory foreign bodies are varied and may depend on the development of the complications. The initial symptoms include odynophagia, dysphagia, globus sensation, dyspnea, coughing, choking sensation, and stridor, and can also be asymptomatic [7]. The duration of the symptoms also is important in suspecting a case of migratory foreign body. Migration of foreign bodies may occur in 24-72 hours [4]. Salting, in 2014, suggested that a case of the migratory foreign body is suspected if there is a suggestive history, positive findings on lateral neck radiography, and a negative finding on rigid endoscopy [7]. Our patient presented with a short history of dysphagia, with positive findings of lateral neck x-ray and negative findings on rigid endoscopy.

The lateral neck X-ray is an inexpensive, easily available tool that can be done to determine the presence and location of the fish bone. However, the neck x-ray is unable to establish if the migration has occurred [7] and it only has sensitivity and specificity of 39% and 79%, respectively, towards diagnosing the ingested fish bones [8]. This is explained by the fact that the fish bones may either appear as radiolucent or radiopaque. CT scan is beneficial in localizing the foreign body and determining the extent of damage done by the migratory foreign body. Besides, the preoperative CT scan also aids the surgeon in mapping the location of vital structures such as major vessels in relation to the foreign body. A 1 mm cut CT scan is the recommended tool to identify thin, small, and radiolucent foreign bodies that cannot be detected in plain radiographs [7]. The surgeon should bear in mind that the exact location of the foreign body may not be similar intraoperatively because the soft tissues of the neck are mobile in relation to the bone and cartilage [9]. Fluoroscopy is another tool that can be used intraoperatively as it provides real-time images of the radiopaque foreign body and it is also beneficial in cases where the foreign body is embedded within the muscles [7]. In general, the gold standard in the diagnosis of migratory foreign bodies is video endoscopy and CT scan of the neck with or without oral contrast [10]. 

An open neck exploration and foreign body removal is the recommended treatment for migratory foreign bodies in the neck to prevent life-threatening complications [6]. There are no certain criteria for the timing of surgery but the general consensus is to perform the surgery as soon as possible after the symptoms have started [11]. Open exploration of the neck to remove the foreign body has been described to be like fishing a needle in the ocean. So, the use of intraoperative fluoroscopy offers a great helping hand, not only in detecting the exact location of the foreign body but also in reducing the surgical time.

## Conclusions

This case report highlights the importance of having a migratory foreign body in the neck as one of the differential diagnoses when there are persistent, unresolved symptoms with the presence of a foreign body on x-ray, despite the failure of endoscopic assessment to detect the foreign body. Diagnosis of the migratory foreign body can be confirmed by a CT scan of the neck and it warrants open neck exploration to remove the foreign body. An open neck exploration together with intraoperative fluoroscopy is the recommended treatment for the migratory foreign bodies in the neck and early surgical intervention is important to prevent life-threatening complications.
